# Alterations in plaque accumulation and gingival inflammation promoted by treatment with self-ligating and conventional orthodontic brackets

**DOI:** 10.1590/2176-9451.20.2.035-041.oar

**Published:** 2015

**Authors:** Mauricio de Almeida Cardoso, Patrícia Pinto Saraiva, Liliana Ávila Maltagliati, Fernando Kleinübing Rhoden, Carla Cristina Alvarenga Costa, David Normando, Leopoldino Capelozza

**Affiliations:** 1 Professor of Orthodontics, Universidade Sagrado Coração (USC), Department of Oral Biology, Bauru, São Paulo, Brazil; 2 Professor of Periodontology, Universidade Sagrado Coração (USC), Department of Oral Biology, Bauru, São Paulo, Brazil; 3 MSc and PhD in Orthodontics, Universidade de São Paulo, School of Dentistry (FOB-USP), Bauru, São Paulo, Brazil; 4 PhD resident in Oral Biology, Universidade Sagrado Coração (USC), Department of Oral Biology, Bauru, São Paulo, Brazil; 5 DDS, Universidade Sagrado Coração (USC), Bauru, São Paulo, Brazil; 6 Adjunct professor, Universidade Federal do Pará (UFPA), Belém, Pará, Brazil

**Keywords:** Corrective Orthodontics, Periodontium, Periodontal index, Dental visible plaque index

## Abstract

**OBJECTIVE:**

The aim of the present study was to evaluate, comparatively, the periodontal response during orthodontic treatment performed with self-ligating and conventional brackets.

**METHODS:**

Sixteen Caucasian individuals of both sexes, aged between 12 and 16 years old and in permanent dentition were selected. Eight individuals were treated with conventional brackets installed on the lower dental arch and self-ligating brackets on the upper arch. Another eight individuals received self-ligating brackets in the lower arch and conventional brackets in the upper arch. The subjects received material and instructions for oral hygiene. Visible plaque index (VPI), gingival bleeding index (GBI) and clinical attachment level (CAL) were evaluated just after installation of orthodontic appliances, and 30, 60 and 180 days later. Mann-Whitney test was used to compare differences between groups (self-ligating and conventional), two-way ANOVA followed by Tukey's test was used to assess CAL at each site of each tooth. Significance level was set at 5%.

**RESULTS:**

No significant changes were found with regard to the assessed parameters (VPI, GBI and CAL) in either one of the systems.

**CONCLUSION:**

No significant changes were found with regard to the periodontal response to orthodontic treatment for the variables assessed and between subjects receiving passive self-ligating and conventional brackets. All individuals had received oral hygiene instructions and had their periodontal conditions monitored.

## Abstract (pt)

**OBJETIVO:**

o objetivo do presente estudo foi avaliar, comparativamente, a resposta periodontal durante o tratamento ortodôntico realizado com braquetes autoligáveis e convencionais.

**MÉTODOS:**

dezesseis indivíduos, leucodermas, em dentição permanente, de ambos os sexos, com idades de 12 a 16 anos, foram selecionados. Oito foram tratados com braquetes convencionais instalados na arcada inferior, e braquetes autoligáveis na arcada superior. Os outros oito indivíduos receberam braquetes autoligáveis na arcada inferior e braquetes convencionais na arcada superior. Os pacientes receberam materiais e instruções sobre higiene bucal. O índice de placa visível (IPV), o índice de sangramento gengival (ISG) e o nível de inserção clínica (NIC) foram avaliados logo após a instalação do aparelho e 30, 60 e 180 dias mais tarde. Para comparar as diferenças entre os grupos (braquetes autoligáveis e convencionais), foi utilizado o teste Mann-Whitney; para analisar o NIC em cada local de cada dente, foi utilizada a análise de variância de duas vias, seguida do teste de Tukey, com nível de significância a 5%.

**RESULTADOS:**

não houve alteração significativa nos parâmetros avaliados (IPV, ISG e NIC), em nenhum dos dois sistemas.

**CONCLUSÃO:**

a resposta periodontal ao tratamento ortodôntico não apresentou diferenças significativas, para nenhuma das variáveis analisadas, entre os indivíduos tratados com braquetes autoligáveis passivos e braquetes convencionais, os quais receberam instruções quanto à adequada higienização bucal e foram submetidos ao monitoramento das condições periodontais.

## INTRODUCTION

After tooth eruption, bracket bonding is considered the second moment of change in the intraoral environment. It can cause qualitative and quantitative changes in the oral microbiota, leading to an increase in the amount of microorganisms not only in saliva, but also in dental plaque.^1^ Dental plaque is the primary etiological factor in the development of gingivitis,^2^ in addition to being the most important factor in the initiation, progression and recurrence of periodontal disease.^3^ Orthodontic brackets might hinder proper oral hygiene, which contributes to the development of an inflammatory process.

Clinically, plaque formation is particularly favored on the cervical surface of brackets, below the leveling arch, and its accumulation is exacerbated by patient's difficulty cleaning these sites. In addition to improper hygiene, gingivitis and gingival hyperplasia are frequently considered the main consequences produced by orthodontic treatment on the periodontium.^4^ When damage caused to the periodontium is considerable, the benefits of orthodontic treatment can be questionable.

Faced with this problem and considering orthodontic brackets as part of its etiology, it would be interesting to discover which parts of orthodontic appliances have the possibility to cause less plaque formation. The advantages of self-ligating brackets include the possibility of performing better hygiene, as they do not require wire ligatures, recognized as the focus of plaque formation. Elastomers are among the ligatures that accumulate a great amount of bacteria,^5^ even elastic ligatures that release fluoride are far from proving effective and reliable in terms of attachment.^6^

Comparing metallic and elastic ligatures, bacteriological findings slightly favor metallic ligatures. Elastic ligatures accumulate 38% more micro-organisms in the form of plaque when compared to metallic ligatures, thereby contraindicating the use of elastic ligatures in individuals with bad hygiene habits.^7^ In terms of bleeding, results were substantially higher with the use of elastic ligatures.^8^ It is worth noting that the more bacterial plaque accumulation, the higher the probability of developing an inflammatory process caused by accumulation and proliferation of bacterial microbiota.^9^

Self-ligating brackets have been a major focus of attention in Orthodontics in recent years, which explains the various designs developed by manufacturers of orthodontic material. All of them have very similar characteristics and can be divided into two groups: active and passive brackets.^10^

In a study conducted by Pellegrini et al,^5^ with the objective of assessing accumulation of bacterial plaque in self-ligating and conventional brackets, the authors concluded that active self-ligating brackets are less likely to accumulate dental plaque when compared to conventional brackets. Nevertheless, it is speculated that active self-ligating brackets allow better hygiene, as they do not have locks or clips completely closing the bracket slot and forming a fourth wall (buccal) similar to molar tubes. Passive brackets, on the other hand, present a buccal wall and, for this reason, could cause plaque accumulation inside the bracket slot.

There is no report of significant difference in the number of bacteria found in self-ligating brackets, compared to conventional ones tied with elastomeric ligatures, whether in metal^14^^,^ 16 or aesthetic brackets.^15^

Depending on the type of brackets used, different microbial trends were found in a study conducted by Mummolo et al.^17^ The authors collected saliva samples from 60 patients, divided into three groups of 20 patients each (self-ligating, conventional and untreated control group) in order to assess *Lactobacillus spp* and *S. mutans*. The assortment of the various species of bacteria change over time during the orthodontic treatment, and seems to show different trends, depending on the type of orthodontic device. Consequently a periodical microbial monitoring using in-office bacteria tests, seems indicated.

All aforementioned considerations, along with the different results found in the studies previously cited and the growing trend towards the use of self-ligating brackets, seem to justify the present study which aims to comparatively evaluate the periodontal response (visible plaque index, gingival bleeding index and clinical attachment level) when orthodontic treatment is performed with self-ligating and conventional brackets.

## MATERIAL AND METHODS

This study was approved by Universidade Sagrado Coração Institutional Review Board (USC 045/11). It comprised 16 Caucasian individuals of both sexes, aged between 12 and 16 years old, selected from a sample of individuals referred to orthodontic treatment in the Department of Orthodontics of the same university. Sample size was calculated by means of BioEstat 5.3 software based on mean and standard deviation values found by a preliminary pilot study. According to this estimation, sample size was determined with a test power of 90%, *α* = 5%, with difference mean and standard deviation values of 1 and 0.9, respectively.

Individuals presenting agenesis or impacted teeth (requiring traction); gingivitis prior to bracket placement; need for orthopedic maxillary expansion, extraction or interproximal wear to reduce tooth size discrepancy; history of use of drugs that induce gingivitis, and patients with skeletal deformities ranging from moderate to severe were excluded from the study. Individuals who agreed to participate in the research answered a questionnaire to detect potential changes in general health and use of drugs.

Another inclusion criterion applied in the study was the presence of complete permanent dentition. Absence of second molars was not considered an exclusion criterion. All participants presented with dental malocclusion and normal skeletal relationships. All selected patients should present, during clinical periodontal examination, a visible plaque index lower than 10% of surfaces (B, MB, DB, L, ML and DL). During clinical examination, the gingival tissue should present a pale pink color without edema, thereby indicating gingival bleeding index equal to zero.^18^

**Figure 1 -:**
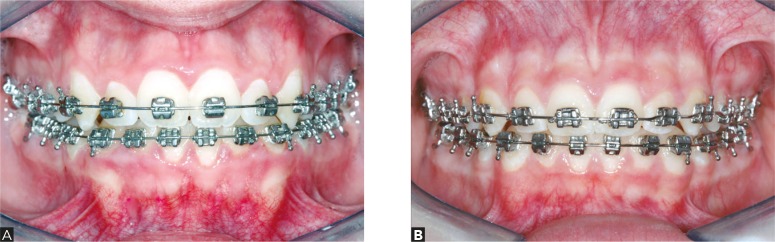
Intraoral photos of a patient in Group 1 (self-ligating brackets in the upper arch and conventional brackets in the lower arch) (A) and Group 2 (conventional brackets in the upper arch and self-ligating brackets in the lower arch) (B).

The individuals were randomly distributed so that eight individuals were submitted to orthodontic treatment with conventional brackets on the lower arch and self-ligating brackets on the upper arch (**Fig 1A**), and eight individuals were submitted to self-ligating brackets on the lower arch and conventional brackets on the upper arch (**Fig 1B**). Since patients simultaneously wore both kinds of brackets, the present study presented acceptable advantages, as there were no alterations in treatment or treatment goals as a result of each type of bracket being placed on different dental arches.

Bracket bonding was performed by a single professional, giving special attention to press the bracket and remove excess resin after achieving final bracket positioning and before the light-curing process. Transbond Plus Color Change (3M, Monrovia, CA, USA) adhesive was used to allow better visualization of excess resin at the time of bonding. Tubes were bonded onto first upper and lower molars in both arches receiving self-ligating and conventional brackets because these teeth were not the object of study.

**Figure 2 -:**
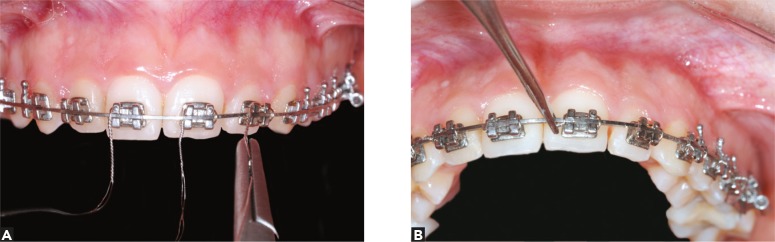
Conventional brackets received metallic ligatures used to tie the arch to the slots (A), always carefully bending them perpendicular to the leveling arch (B) in order to reduce plaque retention

**Figure 3 -:**
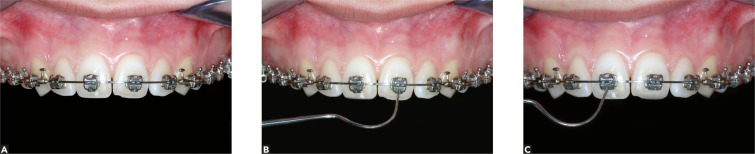
Passive self-ligating brackets present a nickel titanium slot locking mechanism (A), even when a rectangular wire is used (B). The handling for opening and closing the clip was done with the probe #5 (C).

Conventional brackets used were of the Kirium model (Abzil-3M, São José Rio Preto, São Paulo, Brazil), always associated with the use of metallic ligatures to anchor the wires in the bracket slots (**Fig 2**). Passive self-ligating brackets used were of the Portia model (3M, São José Rio Preto, São Paulo, Brazil), with a slot locking mechanism made of nickel titanium (**Fig 3**).

All subjects received a tooth-brushing kit (Oral-B, Procter & Gamble do Brasil). The kit comprised a soft-bristled toothbrush, dental floss and paste. Individuals were also provided with instructions for standardization of oral hygiene and physiotherapy. Toothbrushes and dental floss were changed every two months or whenever necessary. Oral hygiene instructions were given prior to installation of orthodontic appliances, and combined two brushing techniques^19^^,^ 20). Patients were instructed to brush their teeth and use dental floss three times a day.

Patients were assessed by interview and specific clinical periodontal examinations, such as visible plaque index (VPI), gingival bleeding index (GBI) and clinical attachment level (CAL), conducted at six sites per tooth at three different periods (30, 60 and 180 days) after bracket placement. Assessment comprised first and second premolars, canines and central and lateral incisors of each hemiarch, thereby totaling 20 teeth. In order to avoid damage to the participants, all periodontal evaluations and instructions relating to hygiene procedures were given on the same day of orthodontic appliance activation by a single properly calibrated examiner.

Calibration procedures were carried out based on VPI, GBI and CAL of five subjects who were part of the sample,^21^ within seven days.^22^ Clinical evaluation began by observing patients' gingival conditions, using the gingival bleeding index by Löe and Silness.^18^ Subsequently, fucsin-based tablets were used in order to evince accumulated plaque. Ciancio et al's^23^ evaluation parameter index was adopted, as it was specifically developed to assess patients undergoing orthodontic treatment. It considers the buccal surface of teeth, only, as it is subjected to greater dental plaque accumulation after orthodontic corrective appliance installation.

CAL was measured on the buccal, mesiobuccal, distobuccal, lingual, mesiolingual and distolingual faces, with the aid of a manual calibrated periodontal probe (UNC-15). It corresponds to the sum of measurements referring to gingival margin position and probing depth, expressed in millimeters, of each site in each tooth. To calculate this index, each tooth was individually assessed at six different sites and compared at the three assessment periods. GBI was evaluated by visual and compression analysis of gingival soft tissues, according to Löe and Silness.^18^ The scores of each one of the six surfaces of teeth (B, MB, DB, L, ML and DL) were added and the total was divided by six so as to obtain GBI of each tooth. The GBI of each individual was obtained by adding the values ​​of each tooth, with the total divided by the number of teeth evaluated. To obtain VPI, each tooth was individually scored. This index might be estimated for all tooth surfaces or for a few selected sites. For each patient, a mean score of all evaluated teeth was calculated.

Data collected for VPI and GBI were transformed into means and respective standard deviations. To analyze statistical non-parametric ordinal variables, Friedman test was used to detect potential differences among the periods analyzed (30, 60 and 180 days), within the same group. To compare differences between groups (self-ligating and conventional brackets), Mann-Whitney test was used. To assess CAL at each site of each tooth, two-way ANOVA followed by Tukey's test, with significance level set at 5%, were conducted.

## RESULTS

For periodontal evaluation, visible plaque index (VPI), gingival bleeding index (GBI) and clinical attachment level (CAL) were assessed. The analysis of visible plaque index (VPI) compared the values of individuals from the same groups on different examination days. For the self-ligating brackets, no significant differences were found for the mean values between the periods of 30 days (1.76 ± 1.14), 60 days (1.68 ± 0.98) and 180 days (1.48 ± 0.85) (P = 0.4724). Similar results were observed when conventional brackets were analyzed (30 days = 1.78 ± 1.17; 60 days = 1.32 ± 0.72 and 180 days= 1.38 ± 0.68) (P = 0.3480) (**Table 1**). Comparison of visible plaque index (VPI) between groups did not reveal statistically significant results (P > 0.05) in either one of the combinations.

**Table 1 -:** Mean and standard-deviation values of gingival bleeding index (GBI) and visible plaque index (VPI) and p-values for each group.

Indices / Groups	Time				P value
	Initial	30 days	60 days	180 days	
Conventional GBI	1.13 ± 0.83	0.87 ± 0.91	0.53 ± 0.83	0.93 ± 1.03	0.227
Self-ligating GBI	1.13 ± 0.83	0.87 ± 0.99	0.73 ± 0.70	0.73 ± 0.59	0.528
Self-ligating PI	1.99 ± 1.15	1.76 ± 1.14	1.68 ± 0.98	1.48 ± 0.85	0.472
Conventional PI	1.99 ± 1.15	1.78 ± 1.17	1.32 ± 0.72	1.38 ± 0.68	0.348

For the gingival bleeding index (GBI), results were similar to those observed for the visible plaque index (VPI), that is, without statistically significant differences between groups. Indexes observed for self-ligating brackets were: 30 days (0.87 ± 0.99), 60 days (0.73 ± 0.70) and 180 days (0.73 ± 0.59), P = 0.528. As for conventional brackets, values were: 30 days (0.87 ± 0.91), 60 days (0.53 ± 0.83) and 180 days (0.93 ± 1.03), P = 0.227 (**Table 1**). Comparison of gingival bleeding index (GBI) between groups did not reveal statistically significant results (P > 0.05) in either one of the combinations.

Mean probing depth of patients in both groups was 2 ± 0.5 mm. There were no changes in CAL in any of the sites analyzed, nor in any observed periods (P > 0.05), which indicates absence of bone loss. Within 180 days, most subjects presented with recessions and/or gingival hyperplasia not greater than 1 mm, both in the upper and lower arches, regardless of the type of bracket used. The presence of these conditions was not considered statistically significant (P > 0.05).

## DISCUSSION

Numerous studies^6^^,^ 7 ^,^ 8 ^,^ 24 ^,^ 25 ^,^ 26 highlight that orthodontic brackets increase accumulation of dental plaque, which was also demonstrated by the present study. The observation period of the potential effects produced on the periodontium, established by the present study in 30, 60 and 180 days, was considered satisfactory to observe changes in buccal microbiota.^1^ Results reveal visible increase in plaque accumulation and gingival inflammation.^2^^,^ 3 Clinical investigations demonstrate that deleterious effects produced by fixed appliances on the periodontium are caused by insertion loss or by the use of orthodontic bands, which are characterized as ideal sites for bacterial colonization.^25^ In the present study, bands were not used, which limited the deleterious effects produced on the periodontium due to the presence of appliances, different types of brackets, bands and ligatures.

When metallic and elastic ligatures are compared with regard to the amount and quality of bacterial plaque, gingival bleeding index and depth of periodontal bags,^8^ some studies have yielded results that favor the use of metallic ligatures.^6^^,^ 26 For this reason, metallic ligatures were used in the present study. Elastic ligatures accumulate 38% more micro-organisms in the form of plaque in comparison to metallic ligatures.^7^ Still, even metallic ligatures are a focus of plaque formation, which hinders proper hygiene, as proven by the results of the present study. Although elastomeric ligatures present a tendency towards higher dental plaque accumulation in comparison to metallic ligatures, Pandis et al^14^ did not find any differences in the total number of bacteria accumulated in the saliva of patients using conventional brackets with elastomeric ligature and self-ligating brackets. Therefore, elastomeric ligatures do not seem to play a major role in determining salivary and bacterial changes, but influence local adhesion, only.

In a study that allows direct confrontation with the results of the present study, Pellegrini et al^5^ assessed plaque retention during treatment. To this end, the authors installed active self-ligating and conventional brackets with elastomers in 14 dental arches of seven individuals, and concluded that individuals with self-ligating brackets had lower levels of plaque accumulation in comparison to those who received conventional brackets. Between the first and fifth week after bonding, self-ligating brackets presented values of total bacteria and oral streptococcus statistically lower when compared to conventional brackets. These results do not corroborate the present study, which found no differences in plaque formation between the groups treated with self-ligating and conventional brackets, even when a longer observation period was considered (180 days).

Other studies^15^^,^ 16 demonstrate changes in bacterial colonization, especially *S. mutans*, in the period that goes before bracket placement and after analysis. However, there were no differences between self-ligating and conventional brackets. Even though the present research did not aim at analyzing bacterial alterations, the comparison between the aforementioned studies demonstrate that no alterations regarding plaque accumulation and the development of gingival inflammation were found between the two types of brackets used.

Most individuals treated with self-ligating brackets featured a low count of bacteria in bacterial plaque when compared with patients treated with conventional brackets. This is a relevant fact because the acid-producing bacteria that surround and settle in orthodontic appliances are a common problem and cause flaws and discoloration of the tooth enamel surface.^27^ These results suggest that the use of self-ligating brackets predisposes a reduction in dental plaque retention on the tooth surface around these devices. However, against this evidence, no significant differences were found at the site in terms of white lesion development or formation, which depends more on oral hygiene conditions and less on the bracket type or ligature used.^28^

VPI and GBI, calculus index and probing depth were assessed in two types of brackets (conventional and self-ligating) in 50 subjects during 18 weeks. The authors found no differences between the periodontal indexes observed in either one of the groups of brackets.^29^ These results corroborate the data found in the present study, in which comparison of VPI and GBI between the two groups showed no statistically significant differences (P > 0.05).

In this study, most patients, within 180 days, presented with recessions and/or gingival hyperplasia not greater than 1 mm, in both upper and lower arches, regardless of the type of bracket used. This fact was not statistically significant (P > 0.05).

## CONCLUSION

The periodontal response to orthodontic treatment showed no significant differences for either one of the variables when individuals with passive self-ligating and conventional brackets were compared. Importantly, these patients received instructions for proper oral hygiene and were subjected to monitoring of their periodontal conditions.
